# Biological Approaches to Aggressive Cutaneous B-Cell Lymphomas

**DOI:** 10.3389/fonc.2019.01238

**Published:** 2019-11-13

**Authors:** Giulia Tadiotto Cicogna, Martina Ferranti, Annalisa Lazzarotto, Mauro Alaibac

**Affiliations:** Unit of Dermatology, University of Padua, Padua, Italy

**Keywords:** primary cutaneous lymphomas, aggressive cutaneous B cell lymphomas, rituximab, target therapy, new monoclonal antibodies

Primary cutaneous lymphomas (PCLs) are a heterogeneous group of extranodal non-Hodgkin lymphomas (NHLs) that are present only in the skin at the time of diagnosis, without evidence of extracutaneous disease. Among extranodal NHLs, the skin is the second most frequently involved site (30%) after the gastrointestinal system. The incidence rate is estimated at 1 per 100,000 people a year, being primary cutaneous B-cell lymphomas (PCBCLs) only about 25–30% of all PCLs (1).

Biological and clinical features of PCBCLs differ from their nodal counterparts and they are grouped according to their predicted clinical behavior. The indolent forms comprise primary cutaneous marginal zone lymphoma (PCMZL) and primary cutaneous follicle center lymphoma (PCFCL), which usually have an excellent prognosis, despite the high rate of cutaneous recurrences. The aggressive subtypes essentially include primary cutaneous diffuse large B-cell lymphoma leg type (PCDLBCL-LT) and intravascular large B-cell lymphoma (IVLBCL), both characterized by a poor outcome (2). PCLBCL-LT preferentially involves the lower limb with rapidly growing tumors in elderly subjects, whereas IVLBCL is usually widely disseminated in extracutaneous sites at diagnosis, and the clinical appearance of cutaneous lesions may simulate inflammatory skin conditions (3). Consequently IVLBCL is a systemic lymphoma and should not be classified among CBCLs which by definition have no evidence of extracutaneous involvement at the time of diagnosis. The tumoral microenviroment of PCLBCL-LT is characterized by immunosuppressive CD163(+) M2 macrophages and CD33^+^/PD-L1^+^ myeloid-derived suppressor cells (MDSCs) which represent a population of immature myeloid cells with immunosuppressive activity. The presence of these two populations of cells with immunosuppressive properties could be responsible for the poor prognosis of PCDLBCL-LT patients (4).

Selecting the optimal management of PCBCLs is difficult, as they are rare entities and no specific treatment consensus has been stated. Decisions on therapy depend on symptoms, type and stage of the disease and number of lesions. In case of indolent forms, local therapies, such as surgery, radiotherapy or intralesional steroids are appropriate options and overtreatment should be avoided. In subtypes with poor prognosis and in case of dissemination recommended treatment relies on CHOP-like regimens associated with rituximab and local radiotherapy (3). In particular, PCDLBCL-LT still has a high mortality rate, and additional targeted therapies are needed for patients with aggressive cutaneous lymphoma. Gene expression profiling of PCLBCL-LT reveals that this lymphoma subtype is closely related to the activated B-cell–like (ABC) molecular subtype (5). In particular, PCLBCL-LT tends to show frequent recurrence of mutations in genes regulating the nuclear factor kappa B and B-cell signal transduction pathway (6, 7). Furthermore, like primary central nervous system lymphomas/primary testicular lymphomas, a substantial number of PCLBCL-LT harbored translocations involving PD-L1/PD-L2 leading to the upregulation of PD-L1 or PD-L2 within the lymphoma microenviroment (8).

## Monoclonal Antibodies in PCDLBCL-LT

Rituximab has been the first target therapy used in common clinical practice in B cell lymphomas. It is a chimeric anti CD20 monoclonal antibody, binding both normal and malignant B cells expressing this receptor on the surface (9). Rituximab monotherapy was proven effective in primary cutaneous low-grade B cell lymphomas, whereas the ESMO clinical practice guidelines indicate immunochemotherapy with R-CHOP (rituximab, cyclophosphamide, doxorubicin, vincristine, prednisone) as first-line treatment for PCDLBCL-LT (10). On the other hand patients with PCDLBCL-LT are typically elderly, with multiple comorbidities and impaired organ function, which limits therapeutic options in relapsing/refractory cases. The association of rituximab with pegylated liposomal doxorubicin may be an alternative and safer approach and there are preliminary data indicating that this approach is effective and well-tolerated in more aggressive cases with contraindications to CHOP-like regimens (11).

Rituximab monotherapy is generally not adequate and more active and selective anti-CD20 Mabs could be utilized. To this regard currently two categories of anti-CD20 mAbs are undergoing preclinical and clinical evaluation: MAbs with a higher affinity than rituximab with FcγRIIIa, and MAbs with lower immunogenicity. Among these, ofatumumab (anti-CD20) is the human Mab most studied for the treatment of primary cutaneous B-cell lymphomas (9, 12). Moreover, the development of MAbs conjugated with radioisotopes (radioimmunotherapy) represents a very active field of research to improve the efficacy of MAb therapy; two anti-CD20 IgG-radioconjugates, Y-ibritumab, and I-tositumomab, have been approved by the FDA for the treatment of B-cell NHL and could be used in the future for PCDLBCL-LT (13, 14). Associations with other MAbs targeting surface molecules different from CD20 in PCBCL have also shown promising results. To this regard a recent investigation has demonstrated the efficacy of a triple combination of rituximab, lenalidomide and the anti-PD1 inhibitor pembrolizumab in an elderly patient with refractory PCDLBCL-LT (15). Therefore inhibitors of the PD1/PDL1 pathway may represent a potential therapy for PCDLBCL-LT as it appears that unlike PCMZL and PCFCL, PCDLBCL-LT cells express the PDL1 ligand on their surface. The interaction between PD1 and PDL1 decreases T cells proliferation and cytokines production and lead to apoptosis. PDL1-positive tumor cells can bind PD1 expressed on the surface of the tumor infiltrating lymphocytes (TILs) leading them to apoptosis. The presence of PDL1 positive tumor cells in PCDLBCL-LT could explain both the poor prognosis of this tumor and the efficacy of anti PD1/PDL1 therapy in this lymphoproliferative disorder (16, 17).

Two recent developed monoclonal antibodies, lumiliximab and dacetuzumab, are in clinical trials and may have future applications in PCDLBCL-LT. Lumiliximab is a Mab able to bind CD23, a low-affinity IgE receptor found to be higly expressed on chronic lymphocytic leukemia (CLL) cells, whereas dacetuzumab (also known as SNG-40) targets CD40, a molecule expressed on several types of B cells neoplasms (18, 19).

Another promising therapeutic option could be the inhibition of CD33, which is a transmembrane receptor expressed on myeloid cell lines. In the tumor microenvironment this protein can be found in most of the MDSCs and a recent study, demonstrated the presence of these cells in PCDLBCL-LT (4). Humanized antibodies against CD33 have already been used for acute myeloid leukemia and could be used also for PCDLBCL-LT (20).

A highly attractive approach is the application of the bi-specific T-cell engaging (BiTE) antibody (blinatumomab), which targets both CD19 and CD3 antigens. Although the development of this BiTE antibody is currently focused on B-precursor acute lymphoblastic leukemia, promising, early results were also obtained in B-cell lymphomas, as a bridge to allogenic stem cell transplantation in patients with relapsed/refractory DLBCL (21).

## Chimeric Antigen Receptor (CAR) T-cell Therapy

CAR-T cells, which are genetically engineered to express a specific CAR, can recognize tumor-specific antigens and kill target tumor cells. The CD19 protein is specifically expressed on the surface of B-lymphocytes at various stages of development, and more than 95% of B-cell lymphomas express the CD19 antigen. The infusion of CD19-specific CAR-T cells can be efficacious in B-lymphoproliferative disorders (22). However, unlike the encouraging results in B-cell lymphocytic leukemia, the efficacy of anti-CD19 CAR-T cell in the treatment of lymphoma is limited and this may partly be determined by immunosuppressive circuits in tumor microenvironment (23). In order to improve increase CAR-T cell potency, the combination with immune checkpoint inhibitors could be a potential therapeutic strategy. To this regard, a recent investigation has evaluated safety and efficacy of CD19-directed CAR T-cell therapy in combination with the PD-1 inhibitor nivolumab in patients with relapsed/refractory B-NHL (24). This therapeutic strategy might also be a rational and worthwhile approach to treat PCDLBCL-LT.

## Small Molecule Inhibitors

An increasingly interesting target in B-cell malignancies is the Bruton Tyrosine Kinase (BTK) pathway; ibrutinib is the first-in-class among specific BTK-inhibitors. In a case report ibrutinib was administrated to a patient affected with PCDLBCL-LT, after several therapeutic options, with complete remission (25).

Another drug that has shown results in the treatment of PCDLBCL is lenalidomide, an oral immunomodulating agent with several antineoplastic activities, normally used in the treatment of multiple myeloma. In a study of 26 PCDLBCL patients a 20% response rate was observed (26).

Furthermore, development of a great number of new agents to treat NHLs is creating the opportunity to study their efficacy and safety in patients with PCBCL, in particularly in PCDLBCL-LT. To this regard, temsirolimus and everolimus have both demonstrated overall response rates of 30% and a median duration of response of 5.7 months for relapsed systemic DLBCL (27). Moreover, recent studies suggest a potentially promising role of histone deacetilases inhibitors in cases of rapamycin resistance. These studies demostrate that HDAC inhibitors, such as LBH589, may interfere with the mTOR pathway, both at the mTORC1 and mTORC2 level, overcoming mTOR inhibitor resistance in DLBCL (28). Finally patients with DLBCL with overexpression of the BCL2 protein have shown improved response rates combining the BCL2 inhibitor venetoclax with standard chemotherapy (29). The data of the above studies may become useful to tailor therapies for PCDLBCL-LT.

## Conclusions

Several new targeted approaches have been developed in the last years for the therapy of cutaneous lymphomas, with promising results. In particular, for aggressive CBCL management there is still no complete consensus, since until now none of these new drugs has showed complete efficacy and safety. Moreover, despite a safer toxicity profile than standard chemotherapy, these agents continue to present important and still incompletely known toxicities and also there is a need to integrate the achievement of the cure of this group of lymphoproliferative disorders with a good quality of life of the patients and sustainability for health systems.

## Author Contributions

GT, MF, AL, and MA: substantial contributions to the conception of the work, drafting the work and revising it critically for important intellectual content, and approval for publication of the content.

### Conflict of Interest

The authors declare that the research was conducted in the absence of any commercial or financial relationships that could be construed as a potential conflict of interest.

## References

[1] Nicolay J Wobser M. Cutaneous B-cell lymphomas–pathogenesis, diagnostic workup, and therapy. J Dtsch Dermatol Ges. (2016) 14:1207–24. 10.1111/ddg.13164 27992127

[2] Chen S Barnes J Duncan L Primary cutaneous B-cell lymphomas—clinical and histopathologic features, differential diagnosis, and treatment. Semin Cutan Med Surg. (2018) 37:49–55. 10.12788/j.sder.2018.014 29719020

[3] Willemze R Cerroni L Kempf W Berti E Facchetti F Swerdlow SH. The 2018 update of the WHO-EORTC classification for primary cutaneous lymphomas. Blood. (2019) 133:1703–14. 10.1182/blood-2018-11-881268 30635287 PMC6473500

[4] Mitteldorf C Berisha A Pfaltz M Broekaert S Schön M Kerl K. Tumor microenvironment and checkpoint molecules in primary cutaneous diffuse large B-cell lymphoma—new therapeutic targets. Am J Surg Pathol. (2017) 41:998–1004. 10.1097/PAS.0000000000000851 28504999

[5] Pham-Ledard A Prochazkova-Carlotti M Andrique L Cappellen D Vergier B Martinez F. Multiple genetic alterations in primary cutaneous large B-cell lymphoma, leg type support a common lymphomagenesis with activated B-cell-like diffuse large B-cell lymphoma. Mod Pathol. (2014) 27: 402–11. 10.1038/modpathol.2013.156 24030746

[6] Mareschal S Pham-Ledard A Viailly PJ Dubois S Bertrand P Maingonnat C. Identification of somatic mutations in primary cutaneous diffuse large B-cell lymphoma, leg type by massive parallel sequencing. J Invest Dermatol. (2017) 137:1984–94. 10.1016/j.jid.2017.04.010 28479318

[7] Ducharme O Beylot-Barry M Pham-Ledard A Bohers E Viailly PJ Bandres T. Mutations of the B-cell receptor pathway confer chemoresistance in primary cutaneous diffuse large B-cell lymphoma leg-type. J Invest Dermatol. (2019) 139:2334–42.e8. 10.1016/j.jid.2019.05.008 31150604

[8] Zhou XA Louissaint A Jr. Wenzel A Yang J Martinez-Escala ME Moy AP Genomic analyses identify recurrent alterations in immune evasion genes in diffuse large B-cell lymphoma, leg type. J Invest Dermatol. (2018) 138:2365–76. 10.1016/j.jid.2018.04.038. 29857068 PMC6200585

[9] Bello C Sotomayor E Monoclonal antibodies for B-cell lymphomas: rituximab and beyond. Hematology Am Soc Hematol Educ Program. (2007) 2007:233–42. 10.1182/asheducation-2007.1.233 18024635

[10] Willemze R Hodak E Zinzani PL Specht L Ladetto M on behalf of the ESMO Guidelines Committee. Primary cutaneous lymphomas: ESMO clinical practice guidelines. Ann Oncol. (2018) 29:iv30–40. 10.1093/annonc/mdy133 29878045

[11] Fabbri A Cencini E Alterini R Rubegni P Rigacci L Delfino C. Rituximab plus liposomal pegylated doxorubicin in the treatment of primary cutaneous B-cell lymphomas. Eur J Haematol. (2014) 93:129–36. 10.1111/ejh.12315 24635751

[12] Gupta I Jewell R. Ofatumumab, the first human anti-CD20 monoclonal antibody for the treatment of B cell hematologic malignancies. Ann N Y Acad Sci. (2012) 1263:43–56. 10.1111/j.1749-6632.2012.06661.x 22830942

[13] Gupta E Accurso J Sluzevich J Menke D Tun H. Excellent outcome of immunomodulation or Bruton's tyrosine kinase inhibition in highly refractory primary cutaneous diffuse large B-cell lymphoma, leg type. Rare Tumors. (2015) 7:164–6. 10.4081/rt.2015.6067 26788279 PMC4703925

[14] Mancebo S Smith J Intlekofer A Zelenetz A Myskowski P. Treatment response of cutaneous mantle cell lymphoma to ibrutinib and radiotherapy. Clin Lymphoma Myeloma Leuk. (2015) 15:e113–5. 10.1016/j.clml.2014.11.003 25499623

[15] Di Raimondo C Abdulla FR Zain J Querfeld C Rosen ST. Rituximab, lenalidomide and pembrolizumab in refractory primary cutaneous diffuse large B-cell lymphoma, leg type. Br J Haematol. (2019) 187:e79–82. 10.1111/bjh.16211 31566707

[16] Menguy S Prochazkova-Carlotti M Beylot-Barry M Saltel F Vergier B Merlio JP. PD-L1 and PD-L2 are differentially expressed by macrophages or tumor cells in primary cutaneous diffuse large B-cell lymphoma, leg type. Am J Surg Pathol. (2018) 42:326–34. 10.1097/PAS.0000000000000983 29112015

[17] Roschewski M Dunleavy K Wilson W. Diffuse large B cell lymphoma: molecular targeted therapy. Int J Hematol. (2012) 96:552–61. 10.1007/s12185-012-1198-3 23086603

[18] De Vos S Forero-Torres A Ansell S Kahl B Cheson B Bartlett N. A phase II study of dacetuzumab (SGN-40) in patients with relapsed diffuse large B-cell lymphoma (DLBCL) and correlative analyses of patient-specific factors. J Hematol Oncol. (2014) 7:44. 10.1186/1756-8722-7-44 24919462 PMC4065310

[19] Chiappella A Santambrogio E Castellino A Nicolosi M Vitolo U. Integrating novel drugs to chemoimmunotherapy in diffuse large B-cell lymphoma. Expert Rev Hematol. (2017) 10:697–705. 10.1080/17474086.2017.1350164 28665232

[20] Gbadamosi M Meshinchi S Lamba JK. Gemtuzumab ozogamicin for treatment of newly diagnosed CD33-positive acute myeloid leukemia. Fut Oncol. (2018) 14:3199–213. 10.2217/fon-2018-0325 30039981 PMC6331698

[21] Yu J Wang W Huang H. Efficacy and safety of bispecific T-cell engager (BiTE) antibody blinatumomab for the treatment of relapsed/refractory acute lymphoblastic leukemia and non-Hodgkin's lymphoma: a systemic review and meta-analysis. Hematology. (2019) 24:199–207. 10.1080/16078454.2018.1549802 30479190

[22] Hay K Turtle C. Chimeric antigen receptor (CAR) T cells: lessons learned from targeting of CD19 in B-cell malignancies. Drugs. (2017) 77:237–45. 10.1007/s40265-017-0690-8 28110394 PMC5603178

[23] Bao F Wan W He T Qi F Liu G Hu K Autologous CD19-directed chimeric antigen receptor-T cell is an effective and safe treatment to refractory or relapsed diffuse large B-cell lymphoma. Cancer Gene Ther. (2019) 26:248–55. 10.1038/s41417-018-0073-7 30622321

[24] Yaqing C Wenyi L Rui S Xin J Lin C Xiaoyuan H Anti-CD19 chimeric antigen receptor T cells in combination with nivolumab are safe and effective against relapsed/refractory B-cell non-hodgkin lymphoma. Front Oncol. (2019) 9:767 10.3389/fonc.2019.00767 31482064 PMC6709654

[25] Fox LC Yannakou CK Ryland G Lade S Dickinson M Campbell BA. Molecular mechanisms of disease progression in primary cutaneous diffuse large B-cell lymphoma, leg type during ibrutinib therapy. Int J Mol Sci. (2018) 19:E1758. 10.3390/ijms19061758 29899297 PMC6032268

[26] Beylot-Barry M Mermin D Maillard A Bouabdallah R Bonnet N Duval-Modeste AB A single-arm phase II trial of lenalidomide in relapsing or refractory primary cutaneous large B-cell lymphoma, leg type. J Invest Dermatol. (2018) 138:1982–9. 10.1016/j.jid.2018.03.1516 29596904

[27] Witzig T Reeder C LaPlant B Gupta M Johnston P Micallef I. A phase II trial of the oral mTOR inhibitor everolimus in relapsed aggressive lymphoma. Leukemia. (2011) 25:341–7. 10.1038/leu.2010.226 21135857 PMC3049870

[28] Gupta M Ansell S Novak A Kumar S Kaufmann S Witzig T. Inhibition of histone deacetylase overcomes rapamycin-mediated resistance in diffuse large B-cell lymphoma by inhibiting Akt signaling through mTORC2. Blood. (2009) 114:2926–35. 10.1182/blood-2009-05-220889 19641186 PMC2756203

[29] Khan N Kahl B. Targeting BCL-2 in hematologic malignancies. Target Oncol. (2018) 13:257–67. 10.1007/s11523-018-0560-7 29520705

